# Clinical investigation of former workers exposed to asbestos: the health surveillance experience of an Italian University Hospital

**DOI:** 10.3389/fpubh.2024.1411910

**Published:** 2024-06-17

**Authors:** Luigi De Maria, Floriana Pentimone, Domenica Cavone, Antonio Caputi, Stefania Sponselli, Francesco Fragassi, Francesco Dicataldo, Vito Luisi, Giuseppe Delvecchio, Gianmarco Giannelli, Francesco Cafaro, Stefano Sole, Claudia Ronghi, Silvia Zagaria, Giuseppe Loiacono, Gianfranco Sifanno, Giovanni Maria Ferri, Luigi Vimercati

**Affiliations:** University of Bari, Interdisciplinary Department of Medicine, Occupational Medicine Unit, Bari, Italy

**Keywords:** asbestos, health surveillance, formerly exposed workers, Occupational Medicine, asbestosis, mesothelioma

## Abstract

**Background:**

The need for health surveillance of former workers exposed to asbestos was provided by law in Italy after the asbestos ban in 1992.

**Objectives:**

We describe the results of the health surveillance of former workers exposed to asbestos, conducted over 27 years, from 1994 to 2020, at the Operative Unit of Occupational Medicine of the University Hospital of Bari.

**Materials and methods:**

We adopted the health surveillance protocol, which was validated at the national level in 2018.

**Results:**

A total of 1,405 former workers exposed to asbestos were examined. We proceeded with diagnosing pathologies in 339 cases (24% of the cohort subjected to surveillance), with diagnoses of some cases involving multiple pathologies. Specifically, pleural plaques were diagnosed in 49.2% of the 339 cases, asbestosis in 35.9%, malignant pleural mesothelioma (MPM) in 20.3%, mesothelioma of the vaginal tunic of the testis (MTVT) in 9.1%, lung cancer in 5.8%, and laryngeal cancer in 0.8%.

**Conclusion:**

Despite the 1992 asbestos ban, asbestos-related diseases remain a serious public health issue. It is important to establish criteria that ensure the health surveillance of formerly exposed workers minimizes costs, reduces the number of invasive examinations, and optimizes achievable results.

## 1 Introduction

After the issuance of Commission Directive 1999/77/EC of 26 July 1999, the use of asbestos has been completely banned in more than 50 countries, while in others, such as the USA and New Zealand, its use has been greatly reduced (1). Nevertheless, there are still countries, especially developing ones, where this mineral is still used, and an increase in cases of asbestosis and other asbestos-related diseases (ARDs) has been recorded in recent years (2).

At an international level, the amount of asbestos used in compact or friable matrices varies according to the different remediation policies of each country. Estimating the health effects is very complex due to the absence of accurate information on the quantity of asbestos present in each country. Nonetheless, it is estimated that 4.1–7.3 million workers are currently exposed to asbestos (3).

Exposure to products containing asbestos and/or environmental exposure due to residence near natural sources of asbestos fibers or contaminated sites is the cause of the development of ARDs (4). Residential, commercial, and industrial structures that contain asbestos could deteriorate over time or due to natural disasters, causing the dispersion of high levels of suspended asbestos fibers in the air (5). Therefore, attention in the current times should be directed toward these types of exposure instead of occupational ones (6–11).

Notably, there have been over 3,000 recorded uses for asbestos in the past, ranging from fireproof clothing to cigarette filters or fake snow (12). Most users of these products are unaware of their exposure to asbestos. Indirect exposure has often been the cause of ARDs. Parolari, in 2019, described a case of malignant pleural mesothelioma (MPM) in a shoemaker, the repairer of the footwear of employees of a company in the Ledro Valley, where they worked with asbestos (13). The economic industries most involved in exposure to asbestos were steel mills, the engineering, chemical, and petrochemical industries, and construction (14, 15). Other production sectors in which an important use of asbestos has been assessed are the electrical and hydraulic sectors, while the agricultural and printing sectors also deserve attention (16). In other work sectors, forms of exposure are very peculiar. These included seafarers and sailors, who were exposed to the carcinogen, especially when the boat they were sailing on was being repaired (17). Furthermore, asbestos cement was also used instead of asphalt to repair roads (18).

In Italy, from the post-war period until the 1992 ban on asbestos, asbestos was widely used in many production areas. As a result, thousands of workers were exposed, especially in the years between 1950 and 1980 (19–21). In particular, the production of asbestos in Italy at that time was nearly 3.5 million tons, with a production peak recorded between 1976 and 1980 (22).

In recent times, products made of asbestos have been completely banned, and the only permitted work activities that involve exposure to asbestos are remediation, removal, and disposal. The risk of asbestos-related diseases (ARDs) for these work activities should be lower than that in the past, considering the application of preventive and protective measures in the workplace introduced by the most recent directives of the European Union (Directive 2003-18-CE and Directive 2009-148-EC) (23).

In Italy, the need for the health surveillance of former workers exposed to asbestos, already provided for by Legislative Decree 277/91 before the 1992 ban on asbestos, was later reiterated by Legislative Decree no. Lgs. 81/08.

Health surveillance is needed for workers who were included in the register of workers exposed to asbestos during their work. At the end of the employment relationship, the workers underwent a medical examination aimed at receiving information relating to the need for subsequent periodic medical checks, given the long latency between exposure to asbestos and the onset of health effects (24).

Health surveillance is based on a detailed collection of medical history, physical examinations with particular attention to the respiratory system, spirometry examination, diffusion capacity of carbon monoxide tests (DCCM), and radiological examination (chest x-ray). Second-level tests, such as low-dose spiral HRCT or collection of biomarkers such as serum mesothelin and/or serum/plasma osteopontin, which are currently studied for possible future use as predictive factors for the early diagnosis of asbestos-related diseases, are carried out only if deemed necessary (e.g., opacities or signs of fibrosis or pleural effusion at x-ray). The frequency of health surveillance is established on the basis of a risk assessment carried out on a specific case. Given the long latency of asbestos-related diseases, a health surveillance protocol is envisaged for up to 30 years after the end of exposure.

Health surveillance has numerous benefits for workers and communities that range from the possibility of an early diagnosis to the medico-legal aspects, such as a kind of “fast-track” compilation of occupational disease certificates, to the simple collection of clinical-anamnestic data useful for improving knowledge of asbestos-related diseases and counseling activities (25, 26). To achieve this result, an adequate service infrastructure is required. (27, 28).

In Italy, “active” health surveillance has already been implemented in some regions, including Lombardy and Campania. Active surveillance means that the worker is directly contacted to undergo clinical diagnostic tests if already included in the regional lists for the recognition of social security benefits, pursuant to Art. 13 of Law 257/1992, through the National Social Security Institute (INPS) and National Institute of Insurance for Accidents at Work (INAIL) databases. In other regions, “passive” surveillance is carried out only on a voluntary basis and is therefore subject to the request of the individual worker. In most of the regions, health surveillance is entrusted to the Local Health Services (ASL) for the prevention and safety of work environments, with some exceptions: Lombardy identifies the Hospital Departments of Occupational Medicine as responsible, and in Piedmont it is the general practitioner (27).

In this study, we describe the results of health surveillance of formerly exposed workers, conducted over 27 years, from 1994 to 2020, at the Operative Unit of Occupational Medicine of the University Hospital of Bari.

## 2 Materials and methods

Since 1994, at the Operative Unit of Occupational Medicine of the University Hospital of Bari, following the adoption of the health surveillance protocol for formerly asbestos-exposed workers, health surveillance has been carried out on a voluntary basis. The service was provided free of charge with equal opportunity to participate. Inclusion in the study was based both on self-reported exposure or the kind of work task with evidence of working in a well-known sector for asbestos exposure after estimating the exposure in terms of intensity, frequency, and duration.

The health surveillance protocol consists of two phases:

First phase: It is a general evaluation with an assessment of the work and residential history, family, physiological, remote pathology, and recent pathology medical history, followed by a physical examination with particular regard to the respiratory system. Instrumental tests at this stage include spirometry examination, diffusion capacity of carbon monoxide (DCCM) examination, and first-level radiological examination (chest x-ray in two projections). For all individuals with smoking habits, counseling and cessation programs were proposed. All subjects received information on health risks and their rights in the medico-legal and compensation fields.

Second phase: If alterations of probable and/or certain pathological significance were found in phase one (e.g., obstructive, restrictive, or mixed deficit at spirometry and opacities, signs of fibrosis or pleural effusion at X-ray), second-level radiological examinations were performed, such as low-dose computed tomography (LDCT) (29–31), with subsequent consultation by a specialist (pneumology, surgery, and/or oncology). This phase also included the possible determination of reliable, specific, and sensitive biomarkers as an in-depth study for specific cases.

The protocol provided for the double reading of the x-ray and CT scan by a radiologist expert in thoracic pathologies and by a pulmonologist-occupational physician (25, 32). The periodicity of health surveillance, with a new medical examination associated with spirometry and any diagnostic investigations, was established for each subject on the basis of the clinical conditions encountered.

Following the investigations carried out during the visits, in cases where asbestos-related diseases were diagnosed, a certificate of occupational disease was drawn up.

The health surveillance protocol validated by the agreement sanctioned in the Permanent Conference for Relations between the State, the Regions, and the Autonomous Provinces of Trento and Bolzano with protocol 39/CSR of 22 February 2018 was similar to the one implemented at the Preventive Occupational Medicine Unit of Pisa (Cristaudo A. 2006) and adopted in other regions (33–36).

Stata software, STATACORP LLC TEXAS USA, was used for statistical analyses and the construction of frequency tables for the analysis of the distribution of cases and pathologies detected by sex, age, results of the instrumental tests performed, study of the latency, duration of exposure, work activity, and smoking habit. A univariate analysis was conducted using a parametric test, the proportion test, and the *z*-value was calculated (with the associated *p*-value) to study the association between duration of exposure and asbestos-related diseases, as well as the different types of diagnostic approaches. Statistical significance was set at a *p*-value of <0.05.

## 3 Results

From the work history phase, carried out during health surveillance, it was possible to record different potential exposure forms between the subjects. Most of them had work exposure; however, in a smaller number of cases, the exposure was environmental, as they resided near highly contaminated sites or sites known to contain asbestos. The subjects worked in different productive sectors, as shown in Table 5. From 1994 to 2020, a total of 1,405 subjects were examined, of which 1,375 were male (97.8%) and 30 were female (2.1%). They were born between 1911 and 1982 and were divided into four birth cohorts: years 1911–1930, including 117 subjects (8% of the whole group); years 1931–1950, including 816 subjects (58% of the whole group); years 1951–1970, including 450 subjects (32% of the whole group), and years 1971–1982, including 22 subjects (1.5% of the whole group).

For each cohort, the number, age at onset of exposure, duration of exposure, and end of exposure to the carcinogen were assessed (Table 1). Subsequently, the results of the examinations and any pathologies diagnosed during the surveillance checks were assessed.

**Table 1:** Distribution of the cohort by year of birth, sex, average age at the beginning of exposure, and average duration of exposure.

**Birth cohorts (no.)**		**Average age at the beginning of exposure in years (range)**		**Average exposure duration in years (range)**	
**Male**	**Female**	**Male**	**Female**	**Male**	**Female**
1911–30 (117)	-	28 (13–53)	-	25 (1–56)	–
1931–50 (801)	31–50 (15)	24 (15–53)	18 (0–40)^*^	26 (2–57)	19 (5–30)
1951–70 (436)	51–70 (14)	21 (0–45)^*^	9 (0–28)^*^	25 (1–49)	18 (6–34)
1971–82 (21)	71–82 (1)	23 (18–35)	0 (0–0)	11 (4–31)	19 (19–19)
Total (1,375)	Total (30)	24 (0–53)^*^	13 (0–40)^*^	25 (1–57)	18 (5–34)
Total 1,405					

^*^Refers to environmental or family exposures from birth.

Among the female workers subjected to surveillance, 27% were smokers, and among the male workers, 66% were smokers. For the entire cohort of 1,405 formerly exposed workers, regarding the historical period of the beginning of the exposure, 243 subjects (17%) were exposed before 1960, 1,126 subjects (80%) were exposed between 1961 and 1990, and only 36 subjects (2%) were exposed after 1990.

During the health surveillance visits, the formerly exposed workers underwent a respiratory function test that was normal for 911 subjects (64.8%), and in 87 cases, it was not performed due to reduced worker compliance (6.2%). Among the remaining 407 subjects (28.9% of the total subjects examined), an obstructive deficit was found in 230 cases (56.5%), a restrictive deficit in 124 cases (30.5%), and a mixed deficit in 53 cases (13%).

DCCM was normal in 837 subjects (59%) and was not performed in 400 cases (28%), while 11.9% showed alterations, of which 10% were severe alterations.

Chest x-rays showed no anomaly in 941 cases (66.9%), while in the remaining 464 subjects (33.02%), one or more anomalies were reported, including COPD in 39% of the cases, pleural thickening in 20% of the cases, pleural plaques in 14% of the cases, pulmonary nodules and opacities in 10%, and fibrosis in 10% of the cases.

Following the visit and the results of the instrumental tests for 836 subjects (59.5%), it was deemed unnecessary to proceed to the second phase of the health surveillance protocol. A total of 105 subjects, or 7.4% of the subjects under surveillance, had a chest CT scan performed on the prescription of their general practitioner following previously carried out specialist checks outside the health surveillance protocol. In 464 cases (33.02%), a diagnostic examination was carried out as a second-level radiological examination (chest CT scan). Overall, for 569 cases (40.04% of the total), one or more diseases were detected by CT scan, in particular, pleural thickening in 51.4% of cases, pleural plaques in 29.5% of cases, pulmonary nodular opacities in 15.8% of cases, interstitial disease in 13.7% of cases, emphysema in 14.05% of cases, and laryngeal neoplasia in 0.5% of cases (Table 2).

**Table 2:** Distribution of instrumental test results by sex.

		**Male**	**Female**	**Total**	
Spirometry	Mild obstructive deficit	152 (37.6%)	1 (33.3%)	153 (37.6%)	Spirometry
	Moderate obstructive deficit	55 (13.6%)	1 (33.3%)	56 (13.7%)	
	Severe obstructive deficit	21 (5.2%)	-	21 (5.1%)	
	Mild restrictive deficit	70 (17.3%)	1 (33.3%)	71 (17.4%)	
	Moderate restrictive deficit	49 (12.1%)	-	49 (12%)	
	Severe restrictive deficit	4 (0.9%)	-	4 (0.9%)	
	Mild mixed deficit	9 (2.2%)	-	9 (2.2%)	
	Moderate mixed deficit	16 (3.9%)	-	16 (3.9%)	
	Severe mixed deficit	28 (6.9%)	-	28 (6.9%)	
	Total	**404 (100%)**	**3 (100%)**	**407 (100%)**	
DCCM	Mild deficit	95 (57.6%)	2 (66.7%)	97 (57.7%)	DCCM
	Moderate deficit	53 (32.1%)	1 (33.3%)	54 (32.1%)	
	Severe deficit	17 (10.3%)	-	17 (10.1%)	
	Total	**165 (100%)**	**3 (100%)**	**168 (100%)**	
XR	Micronodular nodules/opacities	60 (9.9%)	2 (28.6%)	62 (10.2%)	XR
	COLD	237 (39.2%)	1 (14.3%)	238 (39.4%)	
	Bronchiectasis	5 (0.8%)	-	5 (0.8%)	
	Interstitial diseases/interstitial edema	3 (0.5%)	-	3 (0.5%)	
	Emphysema	23 (3.8%)	-	23 (3.8%)	
	Fibrosis/fibrotic striae	61 (10.2%)	2 (28.6%)	63 (10.4%)	
	Pleural thickenings	118 (19.8%)	1 (14.3%)	119 (19.7%)	
	Pleural plaques	80 (13.4%)	1(28.6%)	81 (13.4%)	
	Pleural effusion	10 (1.6%)	-	10 (1.6%)	
	**Total**	**597 (100%)**	**7 (100%)**	**604** ^*^ **(100%)**	
CAT	Atelectasis	11 (1.4%)	1 (4.2%)	12 (1.5%)	CAT
	COLD	25 (3.2%)	1 (4.2%)	26 (3.3%)	
	Emphysema	79 (10.2%)	1 (4.2%)	80 (10%)	
	Pleural plaques	158 (20.4%)	9 (37.5%)	167 (20.9%)	
	Pleural thickenings	286 (37%)	7 (29.2%)	293 (36.8%)	
	Fibrosis/interstitial disease	75 (9.7%)	3 (12.5%)	78 (9.8%)	
	Bronchiectasis	34 (4.4%)	1 (4.2%)	35 (4.4%)	
	Larynx neoplasm	3 (0.4%)	-	3 (0.4%)	
	Nodules/opacity	89 (11.5%)	1 (4.2%)	90 (11.3%)	
	Pleural effusion	12 (1.5%)	-	12 (1.5%)	
	**Total**	**772 (100%)**	**24 (100%)**	**796** ^**^ **(100%)**	

^*^The totals in the table refer to one or more anomalies simultaneously reported for each subject.

^**^The totals in the table refer to one or more anomalies simultaneously reported for each subject.

CAT, computerized axial tomography; DCCM, diffusion capacity of carbon monoxide; XR, chest x-ray; COLD, Chronic Obstructive Lung Disease.

New diagnoses of pathology were made in 339 subjects (24% of the cohort subjected to surveillance); for some of them, a diagnosis of multiple pathologies was made. Specifically, pleural plaques were diagnosed in 49.2% of the 339 diagnosed cases, asbestosis in 35.9%, MPM in 20.3%, MTVT in 9.1%, lung cancer in 5.8%, and laryngeal cancer in 0.8% (Table 3).

**Table 3:** Distribution of diseases by sex, average latency, and average duration of exposure in years.

		**11–30 years**	**31–50 years**	**51–70 years**	**71–82 years**	**Total**			
						**Male**	**Female**	**M**+**F**	
Asbestosis	N°	**23**	**72**	**27**	**-**	**122**	**-**	**122**	Asbestosis
	Latency^*^	51	42	30	-	41	-	41	
	Exposure^*^	27	26	22	-	25	-	25	
Pleural plaques	N°	**43**	**91**	**33**	**-**	**158**	**9**	**167**	Pleural plaques
	Latency	46	89	84	-	38	53	38	
	Exposure	27	44	49	-	26	19	26	
**MPM**	N°	**2**	**39**	**28**	**-**	**69**	**-**	**69**	MPM
	Latency	65	44	30	-	39	-	39	
	Exposure	30	30	23	-	27	-	27	
MTVT	N°	**-**	**21**	**10**	**-**	**31**	**-**	**31**	MTVT
	Latency	-	40	28	-	36	-	36	
	Exposure	-	23	22	-	22	-	22	
Lung cancer	N°	**3**	**12**	**5**	**-**	**19**	**1**	**20**	Lung cancer
	Latency	62	80	37	-	47	33	46	
	Exposure	36	42	27	-	28	16	27	
Larynx cancer	N°	**-**	**3**	**-**	**-**	**3**	**-**	**3**	Larynx cancer
	Latency	-	44	-	-	44	-	44	
	Exposure	-	26	-	-	26	-	26	
	**Total**	**71**	**238**	**103**	**-**	**402**	**10**	**412** ^**^	

^*^Average latency and average duration of exposure in years.

^**^The totals in the table refer to one or more pathologies diagnosed at the same time for each of the 339 subjects.

MTVT, mesothelioma of the vaginal tunic of the testis; MPM, malignant pleural mesothelioma.

For the 122 diagnosed cases of asbestosis (64% of these were smokers), the average latency was 41 years. Latencies of 38 and 39 years resulted in pleural plaques and MPM among male subjects, respectively. Among the 10 female subjects, 53 years of latency for pleural plaques and 33 for lung cancer were observed among smokers. Among the 19 male subjects with lung cancer and an average latency of 47 years, 11.59% were smokers. Only one smoker was present among the three cases of laryngeal cancer, all with a mean latency of 44 years (Table 3). With regard to the duration of exposure, the 122 cases of asbestosis had an average duration of 25 years, 158 cases diagnosed with pleural plaques had an average duration of 26 years, all among male subjects, and 69 cases of MPM had an average duration of 27 years, all among male subjects. The average duration of exposure for male subjects with lung and laryngeal cancer was 28 and 26 years, respectively. All 31 MTVT cases were also found to have asbestosis.

As shown in Table 4, the majority of diagnoses concerned subjects with an average duration of exposure between 21 and 30 years and a latency in the range between 31 and 50 years, specifically 32% of the cases with asbestosis, 25% of the cases with pleural plaques, 38% of the cases diagnosed with MPM, and 35% of the cases with MTVT.

**Table 4:** Disease distribution by average duration of exposure and latency in years.

	**Average exposure duration in years**	**1–10 years**	**11–20 years**	**21–30 years**	**31–40 years**	**41–50 years**	**51–ω years**	**Total**	
Average latency duration	10–30	4	7	7	3	-	-	21 (17.2%)	Asbestosis
	31–50	6	14	39	13	3	1	76 (62.3%)	
	51–60	1	3	3	9	2	-	18 (14.7%)	
	61–ω	-	-	3	3	1	-	7 (5.7%)	
	**Total**	**11 (9%)**	**24 (19.7%)**	**52 (42.6%)**	**28 (22.9%)**	**6 (4.9%)**	**1 (0.8%)**	**122 (100%)**	
	10–30	2	10	28	9	2	-	51 (30.5%)	Pleural plaques
	31–50	5	14	42	20	7	-	88 (52.6%)	
	51–60	2	3	5	11	1	-	22 (13.2%)	
	61–ω	1	1	3	1	-	-	6 (3.6%)	
	**Total**	**10 (6%)**	**28 (16.7%)**	**78 (46.4%)**	**41 (24.4%)**	**10(6%)**	**-**	**167 (100%)**	
	10–30	1	6	4	1	-	-	12 (17.4%)	MPM
	31–50	2	5	26	11	1	1	46 (66.7%)	
	51–60	-	1	1	4	3	-	9 (13%)	
	61–ω	-	-	1	1	-	-	2 (2.9%)	
	**Total**	**3 (4.3%)**	**12 (17.4%)**	**32 (46.4%)**	**17 (24.6%)**	**4 (5.8%)**	**1 (1.4%)**	**69 (100%)**	
	10–30	1	3	5	-	-	-	9 (29%)	MTVT
	31–50	2	4	11	1	2	-	20 (64.5%)	
	51–60	-	1	1	-	-	-	2 (6.4%)	
	61–ω	-	-	-	-	-	-	-	
	**Total**	**3 (9.7%)**	**8 (25.8%)**	**17 (54.8%)**	**1 (3.2%)**	**2 (6.4%)**	**-**	**31 (100%)**	

MPM, pleural malignant mesothelioma; MTVT, mesothelioma tunica vaginalis testis.

As reported in Table 5, among the 1,385 subjects under surveillance, there were 339 ARDs, equal to 24.5% of cases, and the habit of smoking was present in 65% of cases. Regarding individual occupations, 35% of the subjects were subjected to surveillance, and 21% of these subjects were diagnosed with ARDs. In the largest groups, maintenance workers comprised 19% of this cohort of formerly exposed workers, and among them, 20% had an ARD. The workers of the local asbestos cement factory comprised 11% of the group, with 41% of the ARDs in this group, and welders comprised 11% of the entire cohort, with 25% of the diagnosed ARDs in the group. Regarding the cases with a diagnosis of ARDs distributed in the various occupations, percentages ranging between 33 and 50% of the subjects of each group were glass production workers, subjects with environmental exposure in the workplace, stitchers of jute bags, naval engineers, pipe builders, asbestos cement material (ACM) warehouse workers, tire production workers, and truck drivers. Among the subjects with family exposure or cohabiting with an exposed worker who represented the diffusion source, there were seven cases of pleural plaques among female subjects, all non-smokers and daughters, sisters, or wives of professionally exposed subjects, and the case of a man who was not a smoker with MPM.

**Table 5:** Distribution of occupations by number of smokers, average duration of exposure in years, and diseases.

**Occupations**	**N°**	**No. of smokers**	**Years of average exposure duration**	**Total pathologies**		**Pathologies (number of smokers)**					
				**N°**	**%**	**Asbestosis**	**Pleural plaques**	**MPM**	**MTVT**	**Lung cancer**	**Larynx cancer**
Insulator	498	353	25.6	105	21.1%	44 (31)	41 (28)	27 (19)	11 (8)	8 (5)	0
Maintenance	274	172	25.7	54	19.7%	20 (15)	25 (16)	10 (6)	6 (6)	4 (2)	0
Worker asbestos cement	162	93	22.6	67	41.3%	21 (8)	49 (29)	2 (2)	2 (0)	3 (1)	1 (1)
Welder	153	94	26.5	38	24.8%	13 (8)	13 (10)	14 (9)	3 (1)	1 (0)	2 (0)
Metallurgy manufacturer	45	26	19.6	9	20%	4 (3)	1 (1)	6 (2)	1 (1)	0	0
Electrician	33	21	25.6	4	12.1%	1 (1)	3 (1)	0	0	1 (1)	0
Bricklayer	26	22	31.2	7	26.9%	4 (4)	2 (2)	1 (1)	1 (1)	1 (1)	0
Plumber	24	18	27.9	8	33.3%	1 (1)	6 (6)	1 (1)	0	0	0
Warehouse ACM	21	10	23.8	7	33.3%	2 (0)	4 (1)	1 (0)	1 (0)	1 (1)	0
Industrial cleaner	21	14	19.2	2	9.5%	1 (0)	1 (0)	0	1 (0)	0	0
Family exposure	20	4	18.9	8	40%	0	7 (0)	1 (0)	0	0	0
Naval motorist	14	9	16.7	5	35.7%	3 (3)	1 (0)	2 (1)	2 (2)	0	0
Miscellaneous^*^	11	6	33.4	4	36.4%	1 (0)	3 (3)	1 (0)	0	0	0
Sea worker	10	6	27.5	1	10%	0	1 (1)	0	0	0	0
Railway line manager	9	8	23.5	1	11.1%	1 (1)	0	0	0	0	0
Tire manufacturer	9	4	28.4	3	33.3%	2 (1)	0	1 (0)	0	0	0
Car mechanic	9	4	27	1	11.1%	1 (0)	0	0	1 (0)	0	0
Loading/unloading manager	8	6	26.1	2	25%	0	2 (2)	0	0	0	0
Work, environmental exposure	7	6	19.8	3	42.8%	2 (1)	1 (1)	0	2 (1)	0	0
Painter	7	6	31.7	2	28.6%	0	2 (2)	0	0	0	0
Yarn manufacturer	6	5	24.5	1	16.7%	0	1 (1)	0	0	0	0
Jute bag sewer	5	3	11.4	2	40%	0	2 (0)	0	0	0	0
Cook	5	5	30	1	20%	0	1 (1)	0	0	0	0
Glass manufacturer	4	4	25.2	2	50%	1 (1)	0	2 (2)	0	0	0
Truck driver	3	2	31.3	1	33.3%	0	1 (1)	0	0	0	0
Bulb production manager	1	1	16	1	100%	0	0	0	0	1 (1)	0
**Total**	**1,385**	**902**	**_**	**339**	**24.5%**	**122 (78)**	**167 (106)**	**69 (43)**	**31 (20)**	**20 (12)**	**3 (1)**

^*^Miscellaneous: nurses, asbestos filter winemaker, bankers, land financiers, footwear production workers, booksellers, administrative employees, etc.

MPM, malignant pleural mesothelioma; MTVT, mesothelioma tunica vaginalis testis.

Table 6 shows the association between the duration of exposure and asbestos-related pathologies. Asbestosis, pleural plaques, pleural mesothelioma, mesothelioma of the vaginal tunic of the testis, lung cancer, laryngeal cancer, and all the pathologies considered are significantly associated with exposure periods above the average (25 years). Regarding the association between the duration of exposure and deficits ascertained using pulmonary function tests, mild, medium, and mixed restrictive deficits appear significantly associated with long periods of exposure to asbestos, while obstructive deficits are not. Regarding the association between the duration of exposure and pathologies diagnosed using radiological tests, only pleural effusion confirmed by x-ray appears to be associated with a long duration of exposure, while COPD, emphysema, and fibrosis appear to be associated more with short durations of exposure, probably because they may be linked to etiological factors other than asbestos. The association between the duration of exposure and pathologies diagnosed with computed axial tomography (CAT)—such as COPD, pleural plaques, bronchiectasis, and pleural effusion—shows that these conditions are associated with exposure durations longer than 25 years. The results of the univariate analysis do not take into account other relevant individual and environmental factors, which should be taken into consideration in subsequent analyses on larger cohorts.

**Table 6:** Univariate analysis of the relationship between duration of exposure to asbestos in years, typical asbestos-related diseases, and various types of diagnostic approaches.

		**Total cohort**					**Test**	
		<**25**		≥**25**		**Total**	**Proportion test**	
		***n***	**%**	***n***	**%**	***n***	***z***	***p***
Spirometry	Mild obstructive deficit	70	45.75	83	54.24	153	1.19	0.115
	Moderate obstructive deficit	22	39.28	34	60.71	56	1.7	0.044
	Severe obstructive deficit	8	38.09	13	61.90	21	1.13	0.127
	Mild restrictive deficit	27	38.02	44	61.97	71	2.11	0.017
	Moderate restrictive deficit	17	34.69	32	65.30	49	2.28	0.011
	Severe restrictive deficit	3	75.00	1	25.00	4	−0.79	0.214
	Mild mixed deficit	3	33.33	6	66.66	9	2.3	0.01
	Moderate mixed deficit	10	62.50	6	37.50	16	−1.02	0.153
	Severe mixed deficit	28	100.00	0	0.00	28	#	#
	**Total**	188	38.82	219	61.18	407	3.74	0.001
XR	Micronodular nodules/opacities	30	48.38	32	51.61	62	0.643	0.26
	COLD	137	57.56	101	42.43	238	−2.45	0.007
	Bronchiectasis	2	40.00	3	60.00	5	0.438	0.33
	Interstitial diseases/interstitial oedema	2	66.67	1	33.33	3	#	#
	Emphysema	16	69.57	7	30.43	23	−1.78	0.036
	Fibrosis/fibrotic striae	43	68.25	20	31.75	63	−2.68	0.003
	Pleural thickenings	62	52.10	57	47.89	119	−0.41	0.339
	Pleural plaques	50	61.72	31	38.27	81	−1.6	0.054
	Pleural effusion	1	10.00	9	90.00	10	1.97	0.024
	**Total**	343	56.43	261	43.57	604	−2.91	0.001
CAT	Atelectasis	7	58.33	5	41.67	12	−0.54	0.292
	COLD	7	26.92	19	73.08	26	2.26	0.011
	Emphysema	41	51.25	39	48.75	80	−0.17	0.429
	Pleural plaques	63	37.72	104	61.90	167	3.02	0.001
	Pleural thickenings	157	53.58	136	46.41	293	−1.24	0.107
	Fibrosis/interstitial disease	39	50.00	39	50.00	78	0	0.5
	Bronchiectasis	12	34.29	23	65.71	35	1.8	0.035
	Larynx neoplasm	0	0.00	3	100.00	3	#	#
	Nodules/opacity	38	42.22	52	57.77	90	1.51	0.064
	Pleural effusion	1	8.33	11	91.67	12	2.25	0.012
	**Total**	365	45.66	431	54.34	796	2.18	0.014
Pathologies	Asbestosis	30	24.59	92	75.40	122	4.87	0.001
	Pleural plaques	64	38.10	103	61.67	167	3.02	0.001
	MPM	19	27.53	50	72.46	69	3.39	0.001
	MTVT	8	33.33	23	74.19	31	1.58	0.057
	Lung cancer	7	35.00	13	65.00	20	1.66	0.048
	Larynx cancer	0	0.00	3	100.00	3	#	#
	**Total**	128	31.06	284	68.93	412	7.13	0.001

MPM, malignant pleural mesothelioma; MTVT, mesothelioma tunica vaginalis testis; COLD, chronic obstructive lung disease; **#**, not calculable.

## 4 Discussion

To date, there is a great debate in the scientific literature (24, 27, 37–40) about the usefulness of health surveillance of formerly exposed workers as screening for the early diagnosis of ARDs to improve survival time and the remaining quality of life (20, 24, 30, 41, 42).

The debate also concerns the identification of the workers exposed to asbestos, in particular, whether to include only those with high exposure levels or those with less significant exposure (20). In this regard, the position of the Helsinki Consensus Conference is reported (20, 40): “*We propose that the follow-up of workers highly exposed to asbestos should be continued for at least 30 years from the end of the exposure*.” Therefore, the consensus report “*Asbestos, asbestosis, and cancer. The Helsinki Criteria for Diagnosis and Attribution 2014”* suggested a follow-up of 30 years from the last exposure to asbestos for workers who have been exposed to high doses of the xenobiotic (40). A limitation of our research experience is that we allowed voluntary access by workers already exposed, regardless of exposure quantification, and in any case, allowed access to subjects who had ceased exposure for over 30 years, even those over 75 years old. Despite this, our study shares the well-established aims of health surveillance, such as the recognition of previous exposure, counseling for ARDs and their diagnosis and treatment, the deepening of the causal link by starting the medico-legal insurance process, and providing information and support to mitigate and eliminate additional risks (21).

Our results are in agreement with those of Costantino et al. (43) regarding the working sectors to which the subjects belonged to and the composition of the cohort, which, as expected, consisted mainly of male subjects with occupational exposures (43). The averages of the ages found were similar to the averages we assessed (average age at first access of 58 years, average exposure latency of 39 years, duration of exposure of 25 years, and average age at the beginning of exposure of 23 years). The series concerning the pathological cases was different since Costantino et al. (43) found 37% of patients suffering from asbestosis against our sample of 36%, 40% suffering from pleural mesothelioma against our sample of 20%, and 21% suffering from lung cancer against our sample of 6%. Our data on MPM and MTVT cases distributed in different occupations confirm the widespread use of asbestos in various production sectors and emphasize the need to strengthen the study of any predisposing genetic factors in exposed and formerly exposed subjects. MTVT cases refer to diagnoses made years before the start of our health surveillance and carried out in other regional or national hospital services. The majority of these reported having been treated with radical orchiectomy and radiotherapy without having exhibited a histological report. No cases of peritoneal mesothelioma were detected (44–47).

Regarding the 20 cases of lung cancer (Table 5) found in our study, in 11 cases, 55% of the subjects were also affected by asbestosis; however, asbestosis is not a necessary factor for the development of lung cancer after exposure to asbestos (48). Twelve subjects with lung cancer were also smokers as well as exposed workers. It is known that the combined effect of exposure to asbestos and tobacco smoke on lung cancer risk is more than additive and close to multiplicative; therefore, there is no scientific basis for contrasting these two factors in the risk assessment for individuals with both exposures (12, 48–51). We also observed that the majority of patients with lung cancer associated with asbestosis received a late diagnosis of cancer due to asbestosis-induced masking of symptoms (52). With reference to the occupations and respective production sectors of the 20 detected cases of lung cancer, our data agree with the recent meta-analysis on the impact of occupational exposure to asbestos on lung cancer in Italy (53).

Additionally, in our study, as expected (51), greater diagnostic appropriateness for pleural plaques and dose-dependent pathologies was detected on chest CAT compared to chest X-ray in two projections: 85 diagnoses (14% of total diagnoses) were made by x-ray vs. 168 diagnoses (21%) of pleural plaques made by CT scan.

Unlike Constantino et al. (43), in our study, we recorded four cases with ARDs diagnosed at the age of 40 or less: a 35-year-old chef exposed from the age of 16 years of age, a 39-year-old naval engineer exposed from the age of 18 years, and two male subjects in their 40's, an ACM warehouse worker exposed from the age of 15 years and a glass worker exposed from the age of 21 years. Compared to the data by Cristaudo et al. (25), which reported 23% of pleural plaques, in our study, they accounted for 49% of the pathological plaques. Spirometry was normal for 79% of individuals against our 65%; restrictive deficit was highlighted for 14% of individuals against our 30%, obstructive deficit was reported in 6% of cases against our 56%, and mixed deficit was reported in 1% of cases against our 13%. DCCM was pathological in 26% of the cases compared to 12% of cases in our sample. Regarding thoracic x-rays, 9% of cases underwent this procedure, which is against our 14%.

Our data also show the combined effect of environmental and family exposure on female subjects and the risk of contracting related asbestos diseases (one MPM and seven pleural plaques) (54).

As regards the estimate of exposure to asbestos (certain, probable, and unlikely exposure) in relation to the working sectors, the methodology used is in agreement with that proposed by the IARC, with the scientific literature and with the classification of industrial activities taken from the database of the Italian National Mesothelioma Register (ReNaM) (16, 55).

Nonetheless, using data based on surrogates of the exposure measure, such as job type, can produce misclassifications of the exposures themselves, leading to overestimation or underestimation of the risk (20). Our data referring to the beginning year of exposure and duration of exposure use the duration as a proxy for cumulative exposure (55). Individual exposure to asbestos was assessed retrospectively based on knowledge of the production cycles, as reported by the exposed former workers themselves. The exposures were mainly related to the activities carried out by 17% of the subjects before 1960 and by 80% in the years 1961–1990. Before the 1960's, the dangers of asbestos were not known. In the 30-year period from the 1960's to 1990, there was increasing use of the so-called “miraculous material,” until the ban in the 1990's (40). However, in this study, it was not possible to directly evaluate levels of exposure to asbestos, which is another limitation. However, scientific literature shows that, in work sectors with a known presence of asbestos, exposure was probably high until it was banned, also due to the lack of controls (56, 57).

## 5 Conclusion

In conclusion, despite the 1992 asbestos ban, asbestos-related diseases remain a serious public health issue (58). It is important to establish criteria for health surveillance of formerly exposed workers that allow us to minimize costs, reduce the number of invasive examinations, and optimize results. This result can be achieved through new knowledge shared by the scientific community to update and standardize the protocols (59). Furthermore, diagnostic difficulties may represent a critical factor in the recognition of ARDs as occupational diseases, affecting not only their compensation but also their prognosis and treatment (60).

Both retired workers who were occupationally exposed and the general population need enhanced screening services, timely diagnosis and treatment for ARDs, as well as social and psychological support. In this context, general practitioners can play a fundamental role in the early diagnosis and harmonization of protocols, but this requires continuous training programs (61).

Finally, it is necessary to remember that, in Italy, since 2017, by law, the health surveillance of former workers exposed to asbestos has been recognized as an essential level of assistance and is completely free, with all costs borne by the National Health Service. Given the current state of knowledge, this activity cannot be used for primary or secondary prevention, as no health tests capable of modifying the natural history of asbestos-related diseases are available. Nevertheless, the health surveillance of former workers exposed to asbestos is useful as it allows the history of exposure to be reconstructed, informs subjects about the risks linked to past exposure, and informs about the diagnostic, therapeutic, and medico-legal possibilities for any related diseases.

## Data availability statement

The raw data supporting the conclusions of this article will be made available by the authors, without undue reservation.

## Ethics statement

Ethical approval was not required for the studies involving humans because Ethical approval is not necessary because all medical and instrumental examinations were performed according to Italian laws concerning the protection of workers exposed to occupational risks (D. Lgs. 81/2008). The studies were conducted in accordance with the local legislation and institutional requirements. The participants provided their written informed consent to participate in this study.

## Author contributions

LD: Writing – original draft, Writing – review & editing, Methodology, Formal analysis, Data curation, Supervision, Conceptualization. FP: Writing – review & editing, Writing – original draft, Supervision, Methodology, Formal analysis, Data curation, Conceptualization. DC: Visualization, Project administration, Writing – review & editing, Writing – original draft, Supervision, Methodology, Formal analysis, Data curation, Conceptualization. AC: Writing – review & editing, Writing – original draft, Supervision, Methodology, Formal analysis, Data curation, Conceptualization. SS: Writing – review & editing, Writing – original draft, Supervision, Methodology, Formal analysis, Data curation, Conceptualization. FF: Writing – original draft, Methodology, Formal analysis, Data curation. FD: Writing – original draft, Methodology, Formal analysis, Data curation. VL: Writing – original draft, Supervision, Methodology, Formal analysis, Data curation, Conceptualization. GD: Writing – original draft, Methodology, Formal analysis, Data curation. GG: Writing – original draft, Methodology, Formal analysis, Data curation. FC: Writing – original draft, Methodology, Formal analysis, Data curation. SS: Writing – original draft, Methodology, Formal analysis, Data curation. CR: Writing – original draft, Methodology, Formal analysis, Data curation. SZ: Writing – original draft, Methodology, Formal analysis, Data curation. GL: Methodology, Formal analysis, Data curation, Writing – original draft. GS: Writing – original draft, Methodology, Formal analysis, Data curation. GF: Writing – original draft, Validation, Project administration. LV: Writing – review & editing, Validation, Supervision, Project administration, Methodology, Formal analysis, Data curation, Conceptualization.
